# Influence of Periodic Non-Uniformities of Well-Structured Sapphire Surface by LIPSS on the Alignment of Nematic Liquid Crystal

**DOI:** 10.3390/nano12030508

**Published:** 2022-02-01

**Authors:** Igor Gvozdovskyy, Zoya Kazantseva, Simon Schwarz, Ralf Hellmann

**Affiliations:** 1 Institute of Physics of the National Academy of Sciences of Ukraine, Prospekt Nauki 46, 03028 Kyiv-28, Ukraine; 2 V.E. Lashkaryov Institute of Semiconductor Physics of the National Academy of Sciences of Ukraine, Prospekt Nauki 45, 03028 Kyiv-28, Ukraine; kazants@isp.kiev.na; 3 Applied Laser and Photonics Group, University of Applied Sciences, Würzburger Straße 45, 63743 Aschaffenburg, Germany; simon.schwarz@th-ab.de (S.S.); ralf.hellmann@th-ab.de (R.H.)

**Keywords:** aligning layers, nematic liquid crystals, azimuthal anchoring energy, structured sapphire layers, laser-induced periodic surface structuring

## Abstract

In this study, we report on the alignment properties of nematic liquid crystals on various transparent structured sapphire layers formed by laser-induced periodic surface structures (LIPSS). One-dimensional LIPSS (1D-LSFL) are generated by infrared femtosecond laser pulses along parallel lines covering an area of 5 × 5 mm^2^, with a line spacing that is varied between 7 and 17 µm. These periodic structures, employed as alignment layers, have a spatial periodicity of about 980 nm, a modulation depth of about 100 nm, and exhibit a high quality due to being characterized by a high degree of homogeneity and parallelism of the structured features. It is found that such alignment layers of the sapphire surface lead to a decreasing azimuthal anchoring energy, when the width of the unstructured gap is increased. Modifying the sapphire surface by an ITO-coating with further deposition of a polyimide film increases the azimuthal anchoring energy by a factor of about four up to *W_φ_* ~ 4.25 × 10^−6^ J/m^2^, when the minimum width of the unstructured gap is 7 µm. Comprehensive measurements and comparisons of the azimuthal anchoring energy as well as the pretilt angle for the 1D-LSFL, unstructured gaps, and entire areas depending on the width of unstructured gaps are presented and discussed.

## 1. Introduction

The alignment of liquid crystals (LCs) is an important stage within the production of various LC devices. As a consequence, research on different surfaces as perspective candidates for high-quality alignment of LCs is still continuing. There are a number of various processing techniques allowing to obtain anisotropy on the surface using them as high-quality aligning layers with certain properties for LC orientation [1,2,3,4,5,6,7,8,9,10,11,12,13,14,15,16,17,18].

The most commonly encountered method of orientation of LCs is the rubbing technique, widely used in LCD technology [1,2,3,4]. In addition, in the last two decades, both photoalignment [5,6,7,8,9,10] and the ion/plasma method [11,12] as alternatives to the rubbing technique were successfully studied. Furthermore, uniform alignment methods of LCs were also investigated and proposed in a number of different ways, for instance the usage of Langmuir-Blodgett film [13], e-beam lithography [14], atomic force microscopy (AFM) nano-rubbing [15,16], nano-imprint lithography [17,18], and photolithography [19], respectively.

It is assumed that the homogeneous orientation of LCs is mainly caused by anisotropy created on the substrate surface due to the periodical distribution of grooves, charges, or crosslinking/destruction of the used polymer [1,2,3,4,5,6,7,8,9,10,11,12,13,14,15,16,17,18,19]. Unfortunately, each method of surface treatment referred to above has its own advantages and drawbacks [5]. For the rubbing technique, intensively used in LCD technology, there are some shortcomings, namely accumulation of both the static charges and dust particles [1,5]. Photoalignment is a promising alternative to the rubbing technique [5,6,7,8,9,10], leading to a significant simplification in changing the orientation order of photoproducts under polarized light. However, one of the main shortcomings of the photoalignment technique is, on the one hand, the relatively small areas of treated polymer layers and, on the other hand, a significant degradation of the LCs orientation with time due to the gradual deterioration of the photosensitive polymer layers [5]. Ion/plasma beam treatment as a perspective method leads to the homogeneous planar and tilted orientation of LCs [11,12]. Unfortunately, this technique may result in the unwanted accumulation of static charges, photodestruction of the polymer layer, or deterioration of the polymer properties [5]. In addition, other methods mentioned above [13,14,15,16,17,18,19] show limitations due to the very small areas of processing and limited nano-groove periodicities.

Laser-induced periodic surface structures (LIPSS) became one of the main topics of research interests regarding the interaction of ultra-short pulsed polarized laser light with solid materials, representing a periodic surface texture on a nanometer scale [20,21,22,23,24,25]. There are different types of LIPSS defined by the relation between the nano-grooves spatial period Λ and the applied laser wavelength λ used during the structuring process. Mainly, there are two types of LIPSS, i.e., low spatial frequency LIPSS (LSFL) being characterized by Λ_LSFL_ ≈ λ and high spatial frequency LIPSS (HSFL) with Λ_HSFL_ ≪ λ [24,25,26], while also a more exotic type called supra-wavelength periodic surface structures (SWPSS) with Λ_SWPSS_ > λ is discussed [27,28]. The reason for the creation of LSFL is described by the interference of the electromagnetic field of the laser light and a surface scattered wave generated by the incident laser light [29]. This idea was transferred into a mathematical model by Sipe et al. [30], being the almost widely accepted theory. Moreover, the surface scattered wave was later linked to surface plasmon polaritons [24,31]. The reasons and a fundamental theory for the creation of HSFL, however, are still under discussion [32,33,34].

The formation of LIPSS is a simple and high-speed technique for structuring surfaces which has already previously been demonstrated for LC alignment [35,36,37,38,39]. For the first time, the alignment of the nematic liquid crystal 5CB on a pre-rubbed polyimide layer structured with LSFL was studied in [35], employing a UV Nd:YAG laser with nanosecond pulses. Furthermore, the usage of HSFL generated on a conductive indium-tin oxide (ITO) layer as an aligning layer was studied in [38]. In addition, structuring titanium (Ti) layers with LSFL and the usage as an aligning layer for the orientation of LCs was investigated in [36,37]. Depending on the processing parameters, namely the laser pulse fluence, Φ, and scanning speed, *υ*, the properties of LSFL on titanium aligning layers were studied in [37,39]. It has been shown that the structured Ti-layers additionally modified by polyimide layers can essentially increase the azimuthal anchoring energy (AAE), *W_φ_*, which is one of the main characteristics of the aligning layer [36,37]. Unfortunately, the structured Ti-layers possess a low transparency [39]. Hence, such layers are not appropriate for LC devices working in transmission light.

The creation of LSFL on transparent materials has a practical interest, e.g., in transmission light microscopy for biological and medical applications. Unfortunately, the generation of LSFL on transparent dielectric materials, such as fused silica or sapphire, is a challenging task as compared to metals and semiconductors. The reason is the absence of free charged carriers required to create surface plasmon polaritons on materials in a dielectric environment such as air [40]. However, the generation of LIPSS on transparent materials also occurs owing to the excitation of free charged carriers, formed by multiphoton absorption and avalanche ionization [41,42]. Forming LSFL and HSFL on transparent materials has been studied by several research groups [25,42,43,44,45,46,47,48].

It should be noted that for the application of LIPSS on transparent materials as an alternative aligning technique for the orientation of LCs, along with a high-quality periodicity of structured nano-grooves, as a prerequisite, large areas have to be covered homogeneously by two-dimensional (2D) LSFL or HSFL. However, fabricating high-quality 2D-LSFL with infrared lasers and thus periodicities of about 1 µm has so far predominantly been shown on non-transparent materials, such as metals [21,49]. Only recently, the generation of the 2D-LSFL on transparent dielectric materials has been successfully demonstrated for the first time on sapphire and the influence of the laser scanning direction and laser polarization on the formation of 2D-LSFL was studied by the authors and others [48].

The creation of high-quality LSFL on transparent sapphire allows us to study the structured surface as an aligning layer for the homogeneous orientation of nematic LCs. Unfortunately, the high quality over large areas of the processed sapphire surfaces by LSFL is randomly disturbed in some connecting regions between two neighboring 1D-LSFL lines, as is shown in [48]. This could decrease the uniformity of the LC alignment, leading to an increase of the undesirable and randomly scattered light by the structured sapphire used as aligning layers, and therefore, a decrease of the contrast of LC devices, for example. By avoiding the negative impacts of the random inhomogeneities of structured surfaces as mentioned above, we will study the aligning characteristics of the processed sapphire layer consisting of alternating structured lines and unstructured gaps over large surfaces. In addition, the aligning sapphire layers are additionally coated by an ITO-layer and polyimide film. In this contribution, the dependencies of the pretilt angle, *θ_p_*, and azimuthal anchoring energy, *W_φ_*, as the main characteristics of the aligning sapphire layers and their modifications on the width of the unstructured gaps between two high-quality structured lines are studied.

## 2. Experimental

To study the aligning characteristics of the structured sapphire surfaces, the nematic liquid crystal E7 obtained by Licrystal, Merck (Darmstadt, Germany), was chosen. The optical and dielectrical anisotropy of the nematic E7 at T = 20 °C, λ = 589.3 nm, and *f* = 1 kHz are Δ*n* = 0.2255 (*n*_e_ = 1.7472, *n*_o_ = 1.5217) and Δ*ε* = + 13.8, respectively. Splay, twist, and bend elastic constants of nematic E7 are K_11_ = 11.7 pN, K_22_ = 6.8 pN, and K_33_ = 17.8 pN, respectively [50,51,52]. The parallel and perpendicular components of electric permittivity of the E7 at T = 20 °C are 19.5 and 5.2, respectively [53]. The temperature of the nematic-isotropic transition, T_Iso_, is 58 °C [50].

To obtain planar alignment of the nematic liquid crystal E7, an n-methyl-2-pyrrolidone solution of the polyimide PI2555 (HD MicroSystems, Parlin, NJ, USA) in proportion 10:1 was used.

To examine the aligning characteristics of the structured transparent dielectric material, sapphire substrates (UQG Optics), recently studied in [48], were chosen. The thickness of the sapphire substrates is 2 mm, having an initial surface roughness of about 6 nm.

**Figure 1:**
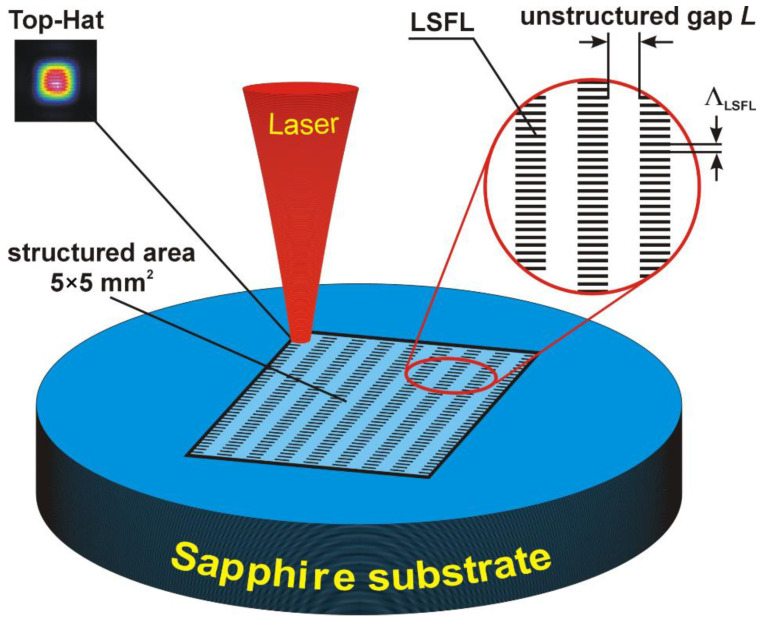
Schematic illustration of the sapphire structuring process. The structured area with a dimension of 5 × 5 mm^2^ has alternating high-quality structured lines (1D-LSFL), with a period Λ = 980 nm and depth A = 100 nm, and unstructured gaps, having a width, *L*, changing within 7–17 μm.

To create LIPSS on sapphire, an ultrashort pulse laser (Pharos, Light Conversion, Vilnius, Lithuania), having a wavelength λ = 1030 nm, a repetition rate of 50 kHz, and a pulse duration of 230 fs (FWHM), was applied. The laser was used in conjunction with a 2D-galvano scanner and a 100 mm f-θ-lens. Furthermore, a diffractive beam shaper (ST-225-I-Y-A, Holo/Or) was applied in the optical setup to transfer the Gaussian beam profile into a Top-Hat profile. The focal spot size of the Top-Hat was measured to have an edge length of l_0_ = 45 µm (1/e^2^), which is shown in Figure 1. The Top-Hat beam profile was used to allow for a more homogenous intensity distribution and thus a higher quality of the generated LSFL, as described in detail elsewhere [54].

For structuring the surface of the sapphire substrates, we used the experimental scheme illustrated in Figure 1. The sapphire substrate was structured by scanning the laser in parallel lines within an area of 5 × 5 mm^2^. The distance between two adjacent scanning lines varied between 30 and 40 µm. LSFL were formed having a line width of 23 µm, and the unstructured gap distance, *L,* between two scanning lines varied between 7 and 17 µm. Please note that a scan distance of 30 µm (7 µm unstructured gap) is the minimum, otherwise, the neighboring lines will be damaged. The anchoring energy (AE) within the width of the unstructured gap of 17 μm may be weak even if strong AE of the 1D-LSFL occurs.

To estimate the value of the AAE *W_φ_* of the structured sapphire surfaces, combined twist LC cells were used [55,56], consisting of tested (structured sapphire) and reference (rubbed PI2555 layer) substrates.

Polyimide PI2555, possessing a strong AAE of *W_φ_* ~ (4 ± 1) × 10^−4^ J/m^2^ [57,58], was used for the preparation of the reference substrates, while the structured sapphire surfaces having various widths of unstructured gaps were the tested substrates. The preparation of the reference substrates with PI2555 films deposited on the glass was detailed in [37,39]. The reference substrates were rubbed in one direction with ten repetitions (*N_rubb_* = 10).

Three types of substrates were tested, namely pure structured sapphire surfaces and two variously modified surfaces. The pure structured sapphire surface is the first type of aligning layer (FTAL), while the structured sapphire surface coated by an ITO layer is the second type of aligning layer (STAL) and the third type of aligning layer (TTAL) has an additional PI2555 layer deposited on the STAL. The PI2555 layer has been formed by the dipping technique using equipment for Langmuir-Blodgett film preparation R&K (Wiesbaden, Germany). For this, STAL was dipped into polyimide PI2555, followed by vertically lifting at a constant speed of about 2 mm/min along the direction of the periodic grooves on the structured sapphire.

Two types of LC cells, namely combined and symmetrical, were used in our studies. To measure the twist angle, *φ,* used for the calculation of the AAE *W_φ_* of the structured sapphire surfaces for all three types (FTAL, STAL, TTAL), combined twist LC cells have been used, described in detail elsewhere [36,37,39]. The easy axis in combined twist LC cells is given by both the direction of rubbing on the reference substrate and the direction of periodic grooves on the tested substrates, having an angle of 45 degrees in relation to each other.

To measure the pretilt angle, *θ*_p_, of symmetrical LC cells, consisting of pair-structured sapphire substrates, we have used the well-known crystal rotation technique [59,60].

The thickness of the studied LC cells was set to 20–25 μm by the Mylar spherical spacer and measured by the interference method, using transmission spectra of empty LC cells. To record the transmission spectra, a spectrometer (Ocean Optics USB 2000, Orlando, FL, USA) was used.

The LC cells were filled with nematic E7 at the temperature T = 61 °C, which is higher than the temperature of the isotropic phase (T_Iso_ = 58 °C) [50], and slowly cooled to the room temperature with a speed ~ 0.1 °C/min in order to avoid possible flow alignment.

In order to analyze the surface topography, a scanning electron microscope (SEM, Phenom ProX, PhenomWorld), an atomic force microscope (AFM, Dimension Icon, Bruker), and a transmitted light microscope (DM6000 M, Leica) were deployed. To measure the twist angle, *φ,* of the combined LC cells in both structured and unstructured gaps for various types of aligning layers, the polarizing microscope BioLar (PZO, Warsaw, Poland) was used.

## 3. Results and Discussion

As mentioned above, in previous manuscripts [36,37,38,39], it has been shown that the periodic structures formed on various metal surfaces having a nano-grooves period of Λ ~ 900 nm and less [38] can be effectively applied as aligning layers.

**Figure 2:**
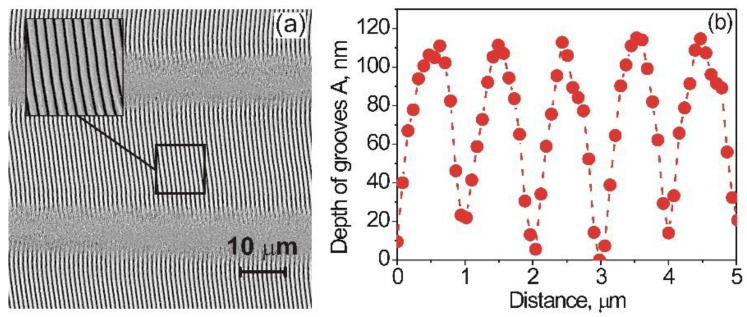
(**a**) SEM image of the sapphire surface with alternating both the high-quality 1D-LSFL with 23 μm and the unstructured gap with 7 μm widths. The portion of the structured line with area 10 × 10 μm^2^ is on an enlarged scale. (**b**) The cross-section of the 1D-LSFL taken with the AFM reveals an average value of the spatial period Λ of 980 nm and a depth, A, of 100 nm.

By varying the main parameters, namely the laser fluence, Φ, scanning speed, *υ,* and the scanning direction relative to the laser polarization, in order to generate structures on the sapphire surface, high-quality 1D-LSFL were experimentally obtained [48]. It was determined that after laser processing of the sapphire surface, both the higher homogeneity and the high parallelism of 1D-LSFL appear, when the scanning direction is the same as the polarization of the laser beam. Here, a scanning speed *υ* = 100 mm/s and laser fluence Φ = 3.16 J/cm^2^ (Φ = E_p_/l_0_^2^, with E_p_ being the pulse energy) are applied. Figure 2a depicts an SEM image of the sapphire surface structured with LSFL. The cross-section of the 1D-LSFL obtained by AFM measurements is shown in Figure 2b. The structured nano-grooves of the 1D-LSFL possess average values of the period Λ of 980 μm and a depth, A, of about 100 nm.

It is well-known that in accordance with Berreman’s theory [3,4], the AE *W_B_* of the aligning layer depends on the depth, A, and period Λ of the nano-grooves and can be written as shown in the equation below:

*W_B_* = 2π^3^∙K∙A^2^/Λ^3^ (1)

where K is the arithmetical mean of the Frank constants (K_11_, K_22_, and K_33_) of nematic LC.

Recently, the estimation of the values of AE *W_B_* of structured layers, possessing a variation of both depth, A, and period Λ, was carried out [37]. It has been shown that a decreasing period Λ or an increasing depth, A, or changing both, leads to an essential increase of the value of AE *W_B_*. Here, the dependence of AAE *W*_φ_ [36,37] or polar anchoring energy (PAE), *W*_θ_ [39], on the depth, A, of the nano-grooves, by changing the scanning speed, *υ,* within a wide range during the laser processing of the metal surface, was experimentally studied. In case of the transparent sapphire substrate, as an alternative aligning surface, the influence of the width, *L,* of the unstructured gap, changing within the range of 7–17 μm, on the value of the AAE *W*_φ_ was examined.

First of all, the value of the AE *W_B_* of the 1D-LSFL was estimated by using Equation (1). By using the average values of period Λ and depth, A, of the 1D-LSFL, the AE *W_B_^struct^* was about 4.5 × 10^−6^ J/m^2^ for the nematic E7. This value of AE *W_B_* is the same as for many other photoalignment surfaces [5]. However, the studied aligning surface consists of alternating high-quality 1D-LSFL and unstructured gaps. Therefore, let us estimate the value of *W_B_^unst^* of the unstructured gaps, by taking into account the fact that surface roughness is about 6 nm, which can be considered as the depth, A, for Equation (1). Let us suppose, hypothetically, that a roughness has a certain periodicity of about 250 nm that is the same average period of grooves, obtained by the usage of traditional methods of LC alignment [3,4,5,6,7,8,9,10,11,12]. Under these conditions, the value of the AE *W_B_^unst^* is about 0.97 × 10^−6^ J/m^2^. It can be assumed that the value of AE *W_B_^unst^* will be much less due to the rather random distribution of surface roughness on the unstructured sapphire gaps, where the conditional periodicity may be much more than 250 nm. Obviously, owing to the fact that LCs are characterized by long-range interaction of the LC molecules in a mesophase, the value of the AE *W_B_^unst^* of unstructured sapphire gaps will depend on both the AE *W_B_^struct^* of two neighboring 1D-LSFL and the width, *L,* of unstructured gaps.

To calculate the value of the AAE *W*_φ_ of the aligning surface for the different types of tested substrates, we used the common method for the combined twist LC cell. According to [55,56], the twist angle, *φ,* is related to the AAE *W*_φ_ according to Equation (2):

(2) $$W\varphi =K_{22}\times \frac{2\times \varphi }{d\times sin2\left(\varphi_{0}-\varphi \right)}$$

where *K*_22_ is the twist elastic constant of nematic E7, *d* is the thickness of the LC cell, *φ*_0_ ≈ 45° is the angle between the easy axes of the reference and tested substrates, and *φ* is the measured twist angle.

**Figure 3:**
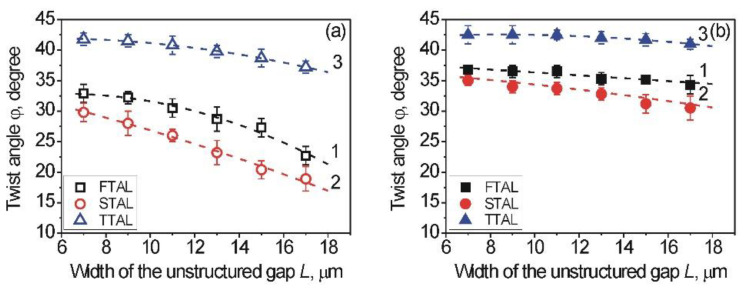
Dependence of the twist angle, *φ*, on the width, *L*, of the unstructured sapphire gaps in case of the FTAL (curves 1, black squares), STAL (curves 2, red circles), and TTAL (curves 3, blue triangles) for: (**a**) unstructured sapphire gaps, located between two 1D-LSFL (opened symbols) and (**b**) 1D-LSFL located nearby the unstructured sapphire gap (solid symbols). Data are average values of the twist angles, *φ*, measured at three different places of certain LC cells and for the three various samples, consisting of sapphire substrates possessing a certain width, *L*, of unstructured gaps. Error bars are absolute deviations of the twist angle from average values. The dashed curve is a guide to the eye.

Since the usage of the 2D-galvano scanner in the structuring process allows to change the width, *L*, of the unstructured gap, the dependence of the twist angle, *φ*, on the width, *L*, was studied between 7 and 17 µm. The twist angle, *φ*, measurements of both 1D-LSFL and unstructured gaps were carried out for the different types of aligning layers under study via the polarizing microscope. The dependence of the twist angle, *φ*, on the width, *L*, of the unstructured gaps is summarized in Figure 3.

As can be seen from Figure 3, the twist angle, *φ,* decreased with increasing width, *L*, of the unstructured gaps in the case of both 1D-LSFL and unstructured gaps for all types of studied aligning layers. However, owing to the appearance of LSFL formed at the structured area of the sapphire surface, the value of the twist angle, *φ*, of the combined LC cell was larger than in the unstructured gaps under the same experimental conditions. In the case of the combined LC cell consisting of a processed sapphire substrate, the measured twist angle, *φ*, was less than the expected angle, *φ*_0_ ≈ 45 degrees, that corresponds to the easy axis, namely an angle between the rubbing direction of the polymer PI2555 film (so-called reference substrate) and the direction of the LSFL on the sapphire substrate (so-called tested substrate). The reason for this deviation of the twist angle, *φ*, from *φ*_0_ is the different value of AAE of the reference and sapphire substrates.

Let us consider the dependence of the twist angle, *φ*, on the width, *L*, for FTAL. As can be seen from Figure 3a for unstructured gaps (curve 1, black opened symbols), the twist angle, *φ*, significantly decreased with the increasing width, *L*, of the unstructured gaps. However, for the 1D-LSFL of the structured sapphire surface, the twist angle, *φ*, slightly decreased with the increasing width, *L* (curve 1, black solid symbols), as can be seen from Figure 3b.

**Figure 4:**
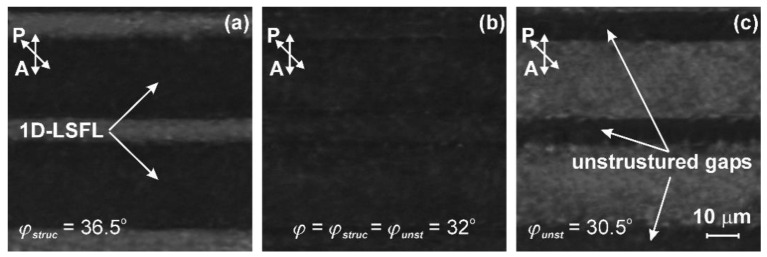
Photographs of twist LC cells under the polarizing microscope placed between a crossed polarizer and an analyzer with the different angles of plane of polarization of the analyzer: (**a**) *φ_struc_* = 36.5 degrees corresponds to the minimum transmittance of 1D-LSFL, (**b**) *φ* = *φ_struc_* = *φ_unst_* = 32 degrees, at which transmittance is the same in both the 1D-LSFL and the unstructured gaps, and (**c**) *φ_unst_* = 30.5 degrees corresponds to the minimum transmittance of the unstructured gaps. The polarization plane of the polarizer coincided with the rubbing direction of the reference substrate having a PI2555 layer. The thickness of the twist LC cell was *d* = 25.5 μm.

Photographs of the 1D-LSFL and unstructured gaps under the polarizing microscope, for the FTAL, possessing an unstructured gap with *L* = 11 μm, are shown in Figure 4. The measurement of the twist angle, *φ*, of the combined LC cell was carried out under the microscope, similar to that used elsewhere [37,39]. For the twist LC cell consisting of the tested substrate with FTAL, at a value of *φ_struc_* = 36.5 degrees, the minimum transmittance of the 1D-LSFL was observed (Figure 4a). The rotation of the analyzer (A) at an angle of 32 degrees resulted in the same transmittance of the FTAL at both the 1D-LSFL and the unstructured gaps, as can be seen in Figure 4b. The minimum transmittance of the unstructured gaps of the FTAL was observed at a twist angle *φ_unst_* = 30.5 degrees (Figure 4c).

It can be assumed that the main reason for the difference of the measured twist angles between the two lines is the nano-grooves on the 1D-LSFL, possessing a stronger AAE *W_φ_* than the unstructured gaps. This is in good agreement with previous estimations made by Berreman’s theory [3,4]. The same behavior was also observed for the modified sapphire surfaces created by both the ITO-coating (curves 2, red circles) and the deposition of polyimide PI2555 (curves 3, blue triangles), as can be seen from Figure 3.

As Figure 3 shows for the STAL (curves 2, red circles), consisting of a structured sapphire substrate coated with ITO, the twist angle, *φ*, of the combined LC cell is smaller than in the case of the FTAL (curves 1, black squares). It is worthwhile to note that the planar (P) alignment of nematic E7 for the combined LC cell, consisting of the test substrate with STAL, is unstable and changes over time to the homeotropic (H) alignment. The planar–homeotropic (P-H) transition lasted a total of two hours. Recently, the same P-H transition of two nematics (E7 and MLC-6609) was observed for the aligning layer, consisting of the structured Ti-layer processed by the laser structuring method and coated with ITO [39]. The twist angle measurements were carried out 30 min after the cooling of the LC cells, filled by nematic E7.

Using the TTAL, consisting of the STAL coated with polyimide PI2555, resulted in a substantial increase of the twist angle, *φ,* for both the unstructured gaps (curve 3, blue opened triangles) and 1D-LSFL (curve 3, blue solid triangles), as shown in Figure 3. In the case of the 1D-LSFL, the twist angle, *φ,* of the combined LC cell shows its maximum value. The main reason for this maximum value is the PI2555 layer, possessing a strong AE, while the homogeneous alignment was observed due to the laser structuring of the initial sapphire substrates, which is comparable to the rubbing process in the literature [57,58]. As shown in [36,37,39], the periodically nano-structured Ti surfaces coated with PI2555 have a strong AE even if they are used without unidirectional rubbing.

**Figure 5:**
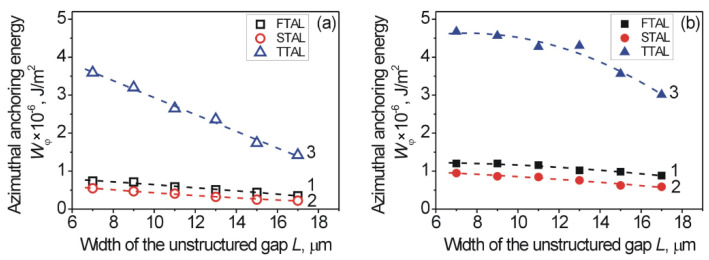
Dependence of the AAE *W_φ_* on the width, *L*, of the unstructured gaps in case of the FTAL (curves 1, black squares), STAL (curves 2, red circles), and TTAL (curves 3, blue triangles) for: (**a**) unstructured sapphire gap, located between two 1D-LSFL (opened symbols), and (**b**) 1D-LSFL that is located next to the unstructured sapphire gap (solid symbols). The dashed curve is a guide to the eye.

To calculate the AAE *W_φ_* of the aligning layer, we used the measured values of the twist angle, *φ*, of the combined LC cells and Equation (2). The dependence of the AAE *W_φ_* on the width, *L*, of the unstructured gaps for the different types of studied aligning layers in both unstructured gaps (opened symbols) and 1D-LSFL (solid symbols) is shown in Figure 5.

As mentioned above, small values of the twist angle, *φ*, of the combined LC cells are caused by, on the one hand, weaker AAE *W_φ_* of the FTAL (black squares, Figure 5) and STAL (red circles, Figure 5), and on the other hand, due to the increase in the width, *L*, of the unstructured gaps. In addition, the absence of nano-grooves also results in a decrease of the twist angle, *φ*, and consequently to a smaller value of AAE *W_φ_*. Using the polyimide PI2555 layer for the modification of the ITO-coated structured sapphire surfaces, the STAL (i.e., TTAL was formed) leads to an increase of the AAE for both the unstructured gaps (opened blue triangles, Figure 5a) and the 1D-LSFL (solid blue triangles, Figure 5b).

**Figure 6:**
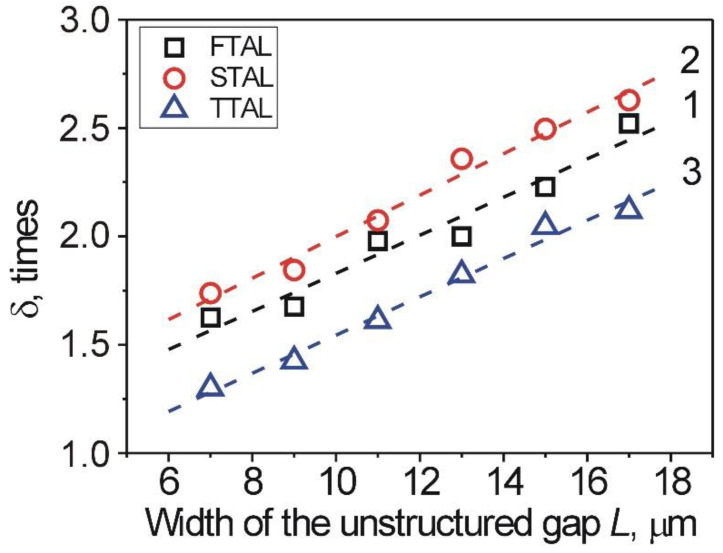
Dependence of the ratio *δ* between the AAE *W_φ_* of the 1D-LSFL and unstructured gaps on the width, *L*, of the unstructured gap for: (1) the FTAL (opened black squares), (2) the STAL (opened red circles), and (3) the TTAL (opened blue triangles). The dashed line is a guide to the eye.

It is worthwhile to note that the unstructured gaps also have relatively large values of AAE *W_φ_*, in comparison to the 1D-LSFL. Taking into account the estimations of the AE *W_B_*, carried out above on the basis of Berreman’s theory [3,4], it can be seen that for the 1D-LSFL, the value of *W_B_^struct^* was about 5 times higher than the value of *W_B_^unst^* in the case of the unstructured gaps. However, for the experimentally obtained values of AAE *W_φ_* (Figure 5), the ratio *δ* between the 1D-LSFL and unstructured gaps was small and depends on both the type of aligning layer and width, *L*, of the gap, as can be seen from Figure 6.

The minimum ratio *δ* was observed for the TTAL (opened blue triangles), while for the STAL (opened red circles) the ratio revealed the highest values over the entire range. In addition, the increase of the width, *L*, leads to an increase of the ratio *δ* caused by the decrease of the AAE *W_φ_* for the unstructured gaps (Figure 5). Consequently, the lower the ratio *δ* between the AAE *W_φ_* of the 1D-LSFL and unstructured gaps, the better the aligning characteristics of the studied layer. Assuming that this ratio *δ* → 1, the AAE *W_φ_* will be the same in both the 1D-LSFL and unstructured gaps. Thus, the presence of unstructured gaps with a small width, *L,* will not affect the alignment properties of the entire area of the layer. With the ratio *δ* ≫ 1, a weak AAE *W_φ_* of the unstructured gaps possessing a larger width will be observed. The increase of the width, *L,* of the unstructured gaps has an influence on the alignment properties of the entire area of the aligning layer.

**Figure 7:**
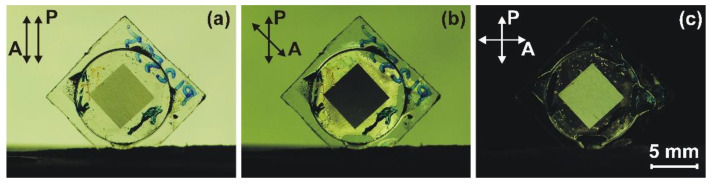
Photographs of the twist LC cell placed between a pair of polarizers with different angles of planes of polarization: (**a**) parallel, (**b**) twist angle *φ* ≈ 33.5 degrees, and (**c**) perpendicular. The LC cell consists of the reference (rubbed PI2555 layer) and tested substrate, having the structured sapphire surface with 1D-LSFL (23 μm width) and unstructured gaps (11 μm width). The twist angle, *φ^Σ^*, of the processed area of the LC cell is about 33.5 degrees. The thickness of the twisted LC cell is *d* = 24.6 μm. The rubbing direction of the reference substrate (PI2555 layer) coincides with the plane of polarization of the polarizer, while the analyzer can rotate freely.

The general appearance of the combined LC cell in crossed polarizers with the different polarization angles of the analyzer is shown in Figure 7. The measurement of the twist angle, *φ^Σ^*, for the entire area of 5 × 5 mm^2^ was carried out by the scheme described in [37], by rotating the analyzer to an angle in which the minimum transmittance of the entire 1D-LSFL area (Figure 7b) is observed.

**Figure 8:**
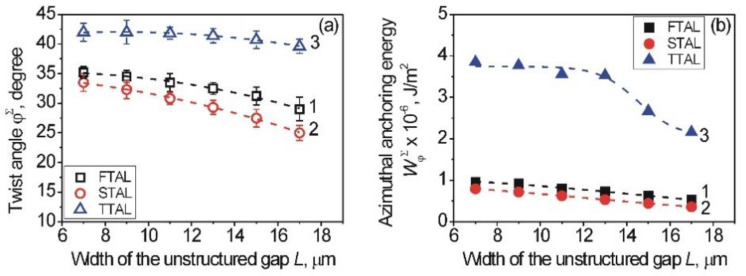
Dependence of the twist angle, *φ^Σ^* (**a**), and AAE *W_φ_^Σ^* (**b**) of the entire area on the width, *L*, of the unstructured sapphire in the case of the FTAL (curves 1, black squares), STAL (curves 2, red circles), and TTAL (curves 3, blue triangles). Entire area of structured sapphire surface consists of the unstructured sapphire gap, located between two 1D-LSFL. The dashed curve is a guide to the eye.

Therefore, the twist angle, *φ^Σ^*, measured for the entire area of the aligning layer included alternating both the 1D-LSFL and unstructured gaps as a function from the width, *L,* as shown in Figure 8a. By comparing the twist angles of the entire area with each individual area (i.e., 1D-LSFL and unstructured gap), it can be seen that the twist angle, *φ^Σ^*, of the entire area is smaller than the twist angle, *φ,* for the 1D-LSFL, and vice versa it is greater than the twist angle, *φ*, for the unstructured gaps.

For each type of aligning layer, the calculated value of AAE *W_φ_^Σ^* of the entire area of the structured surface of the twist LC cells is shown in Figure 8b, again for the different width, *L*. For each type of aligning layer, the *W_φ_^Σ^* has a higher value, but less than that for a single 1D-LSFL (Figure 5b). As can be seen from Figure 8b, the AAE *W_φ_^Σ^* can be changed by changing the width, *L*, of the unstructured gap, which is similar, for example, to changing the number of unidirectional rubbings, *N_rubb_*, during the rubbing technique [57,58,61], the irradiation time of photopolymers in the process of photoalignment [5,55,56], or the scanning speed, *υ*, and the laser pulse fluence, Φ, when examining nano-structured metal surfaces for the alignment of LCs [36,37,38,39].

As can be seen from Figure 8a, the usage of PI2555-coating leads to, on the one hand, an increase AAE *W_φ_^Σ^* in comparison with the entire area of FTAL and STAL, and on the other hand, slight changes of the value of AAE *W_φ_^Σ^* with increased width, *L,* of the unstructured gaps; namely, the AAE *W_φ_^Σ^* is changing within the range of 3.53–3.85 × 10^−6^ J/m^2^ when changing the width, *L,* of the unstructured gaps in the range from 7 to 13 μm.

It is worthwhile to note that for TTAL possessing various widths, *L*, of the unstructured gaps, the maximum values of AAE *W_φ_^Σ^* are approximately the same as for AAE of pre-rubbed polyimide [35] or ITO [38], where the entire area of the surface is covered with nano-grooves. It is obvious that changing the processing parameters (wavelength, λ, and laser fluence, Φ) can lead to an increase of the AAE of the sapphire aligning layers with non-uniformities [35,37]. However, a stronger AAE of the surface was obtained by using titanium layers at certain processing parameters (laser fluence, Φ, and speed of scanning, υ) by LIPSS and further covering them with polymers [36,37,39]. It should be emphasized that in comparison with sapphire surfaces, the titanium aligning layers possess low transparency that may limit their use to LC devices working on transmission of light. It should be further noted that as can be seen from Figure 8b in the case of structured sapphire surfaces having various widths, *L*, of the unstructured gaps in the range of 7–13 µm, the value of AAE *W_φ_^Σ^* is roughly the same. It is a new and important result because aligning surfaces treated with this method can be obtained without changing surface alignment characteristics (e.g., AAE *W_φ_^Σ^*, direction of the easy axis of alignment, twist, *φ^Σ^*, and pretilt, *θ^Σ^_p_*, angles) in a shorter time than in the case of structuring entire areas [35,36,37,38,39].

Recently, it has been shown that the pretilt angle, *θ_p_*, can depend on the exposure of photoalignment layers [62] or scanning speed, *υ,* while structuring titanium layers by laser-processing the surface [39]. It is obvious that, as shown above, apart from the influence on the value of AAE *W_φ_*, changing the width, *L,* of the unstructured gaps will also affect the pretilt angle, *θ^Σ^_p_*, of the LC molecules.

Let us consider the influence of the width, *L,* of unstructured gaps on the pretilt angle, *θ^Σ^_p_*, of the symmetrical LC cell, consisting of a pair of similar substrates with a certain type of aligning layer. The measurements of the pretilt angle, *θ^Σ^_p_*, of the symmetrical LC cells were carried out by the crystal rotation technique [59,60]. It should be noted that in the case of the symmetrical LC cell, consisting of a pair of substrates with STAL, the measurement of the pretilt angle, *θ^Σ^_p_*, was carried out after each hour, because in the case of the combined twist LC cell, consisting of the tested substrate with STAL, we observed the P-H transition, as described above.

**Figure 9:**
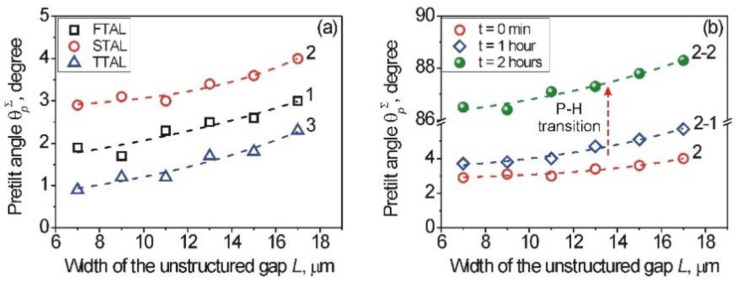
(**a**) Dependence of the pretilt angle, *θ^Σ^_p_*, on the width, *L*, of the unstructured sapphire gap for symmetrical LC cells, consisting of a pair of substrates, possessing the FTAL (curve 1, opened black squares), STAL (curve 2, opened red circles), and TTAL (curve 2, opened blue triangles). (**b**) Dependencies of the pretilt angle, *θ^Σ^_p_*, on the width, *L*, of the unstructured sapphire gap for different storage times of the symmetrical LC cell, consisting of the STAL (structured in a certain manner, sapphire surface coated with ITO layer): t = 0—curve 2 (opened red circles); t = 1 h—curve 2-1 (opened blue diamonds); t = 2 h—curve 2-2 (solid green spheres).

The dependence of the pretilt angle, *θ^Σ^_p_*, of the entire processed area for symmetrical LC cells on the width, *L*, of the unstructured gap is shown in Figure 9.

Figure 9a shows an increasing pretilt angle, *θ^Σ^_p_*, with the increasing width, *L,* as expected, because the AAE *W^Σ^_φ_* is weakening with the increasing width, *L* (Figure 8). The pretilt angles, *θ^Σ^_p_*, are changing within the range of about 1–3.5 degrees, which is common for all aligning surfaces possessing the planar (P) alignment of LCs [5]. In comparison with the FTAL (curve 1, opened black squares) and the STAL (curve 2, opened red circles), for the TTAL (curve 3, opened blue triangles), the values of the pretilt angle, *θ^Σ^_p_*, were smaller due to the presence of the PI2555 layer that possesses a stronger AAE *W^Σ^_φ_*. However, it should be noted that in the case of the STAL, the pretilt angle, *θ^Σ^_p_*, was unstable. The change of the pretilt angle, *θ^Σ^_p_*, evolving with the storage time of the LC cell, consisting of the pair of substrates having the STAL, is shown in Figure 9b. It can be seen that after 2 h storage of the symmetrical LC cell, the H orientation of the LC molecules was observed (curve 2-2, solid green spheres, Figure 9b). The pretilt angle, *θ^Σ^_p_*, increased to 86–89 degrees depending on the width, *L,* of the unstructured gaps. It should be noted that a similar P-H transition for the nano-structured Ti-layer coated with the ITO layer, studied recently as an aligning layer, was also observed [39]. It can be seen from Figure 9 that the use of structured sapphire with different gap widths, *L*, modified by various material layers allows us to control the pretilt angle, *θ^Σ^_p_*, in the planar alignment or to change from the planar to homeotropic alignment.

## 4. Conclusions

We studied the alignment properties of transparent dielectric sapphire as an aligning layer, since the usage of transparent dielectric materials for the alignment of LC is important in the production of LC devices, operating on the principle of light transmission. To obtain a large area of the aligning surface, the transparent sapphire was processed by alternating structured gaps, revealing high-quality 1D-LSFL at a constant width of 23 μm as well as unstructured gaps between these 1D-LSFL, having a width which was investigated within 7–17 μm. The dependence of the azimuthal anchoring energy, *W_φ_*, and pretilt angle, *θ_p_*, as important aligning characteristics on the width, *L*, of the unstructured gap of the sapphire surface was experimentally studied. The value of azimuthal anchoring energy can be controlled in two different ways, namely, on the one hand, by modification of the structured sapphire surface using different materials, e.g., an ITO or polymer layer, and on the other hand, by changing the width, *L*, of the unstructured gaps. It was shown that azimuthal anchoring energy of both the 1D-LSFL and the unstructured gaps and also the entire processed area depends on the width, *L*, of the unstructured gaps.

It was further experimentally shown that the value of azimuthal anchoring energy of the structured sapphire surface is of the order of photoalignment layers. Furthermore, a dependence of the azimuthal anchoring energy and the pretilt angle, *θ_p_*, on the width of the unstructured gaps was found. To change the value of the azimuthal anchoring energy, the structured sapphire surface was also coated by an ITO and a polymer layer. Polymer coatings of the structured sapphire surface increased the value of the azimuthal anchoring energy by about four times. The use of an ITO layer leads to a significant change of the pretilt angle due to the unstable planar orientation of nematic E7, resulting in the planar–homeotropic alignment transition. It was experimentally obtained that an increase of the width of the unstructured gaps leads to a decrease of the azimuthal anchoring energy, while the pretilt angle slightly increased. However, the gain of azimuthal anchoring energy can be resolved by changing the structuring process conditions of transparent dielectric materials that will lead to a decrease of the period and/or increase of the depth of nano-grooves.

## Data Availability

The data presented in this manuscript can be obtained from the corresponding author upon request.
